# Motivated explanation

**DOI:** 10.3389/fnhum.2015.00559

**Published:** 2015-10-16

**Authors:** Richard Patterson, Joachim T. Operskalski, Aron K. Barbey

**Affiliations:** ^1^Department of Philosophy, Emory UniversityAtlanta, GA, USA; ^2^Decision Neuroscience Laboratory, Beckman Institute for Advanced Science and Technology, University of Illinois at Urbana–ChampaignUrbana, IL, USA

**Keywords:** explanation, motivation, abductive reasoning, causal inference, inference to the best explanation

## Abstract

Although motivation is a well-established field of study in its own right, and has been fruitfully studied in connection with attribution theory and belief formation under the heading of “motivated thinking,” its powerful and pervasive influence on specifically explanatory processes is less well explored. Where one has a strong motivation to understand some event correctly, one is thereby motivated to adhere as best one can to normative or “epistemic” criteria for correct or accurate explanation, even if one does not consciously formulate or apply such criteria. By contrast, many of our motivations to explain introduce bias into the processes involved in generating, evaluating, or giving explanations. Non-epistemic explanatory motivations, or following Kunda's usage, “directional” motivations, include self-justification, resolution of cognitive dissonance, deliberate deception, teaching, and many more. Some of these motivations lead to the relaxation or violation of epistemic norms; others enhance epistemic motivation, so that one engages in more careful and thorough generational and evaluative processes. We propose that “real life” explanatory processes are often constrained by multiple goals, epistemic and directional, where these goals may mutually reinforce one another or may conflict, and where our explanations emerge as a matter of weighing and satisfying those goals. We review emerging evidence from psychology and neuroscience to support this framework and to elucidate the central role of motivation in human thought and explanation.

## Introduction

Human beings are powerfully motivated to understand the nature, history, and future direction of their environment. Many of our purposes motivate us to *get the correct or accurate explanation* of a situation or event (*meet relevant epistemic norms of explanation)*; others motivate us to *arrive at some preferred explanation*, where our preference derives from non-epistemic goals—i.e., goals other than that of accuracy or meeting epistemic norms (Kruglanski, 1980; Pyszczynski and Greenberg, 1987; Chaiken et al., 1989; Kunda, 1990). The latter goals include self-justification, attainment of emotional satisfaction, bringing about interpersonal reconciliation, reducing cognitive dissonance, amusing ourselves, assigning or avoiding blame, and many more. These goals and motivations we will call “directional,” or simply “non-epistemic.”

It is clear that multiple motives can be at play simultaneously, and these may work in concert to support a search for the epistemically best or most accurate explanation, or they may conflict, with some urging us toward correctness, others toward a particular explanatory result that serves important directional purposes (i.e., those other than satisfying norms of correctness or accuracy). When our explanatory goals compete we may try to find an explanation that at least partially satisfies them all, or we may choose to satisfy some and ignore others. Our motives in seeking an explanation, whether they be one or many, mutually reinforcing or directly in competition, potentially influence all the processes involved in generating, evaluating, accepting, or giving explanations.

How, then, given that most events of interest might in principle be explained in different ways, do we decide on one possible explanation rather than another? This way of framing the question reflects our focus here on the process of abduction, or “inference to the best explanation.” In essence, abduction differs from deductive and inductive inference in that it takes the fact that some candidate explanation appears to be the best explanation (in the sense of meeting epistemic norms, or standards of correctness) as at least partial grounds for thinking that it is the correct explanation. Such inferences are ubiquitous in everyday life and important in science as well (Salmon, 1989). In this article, we propose a theory of motivated explanation that characterizes the role and influence of motivation on human attempts to find “the best” explanation of a given phenomenon.

The Section entitled “Explanatory Processes” identifies three core processes involved in *generating* what one takes to be the most accurate, or epistemically best, explanation and six processes involved in *evaluating* explanations for accuracy (See Table 1 for a brief explanation of each). All of these processes are “points of vulnerability” (to borrow a phrase from Redish et al., 2008; Redish, 2013) to biases, heuristics, and in specific circumstances, directional motivational influences. We note several points at which these two sets of processes overlap, in the sense that some of them play a role in both generation and evaluation of explanations. The Section entitled “To Meet, or Not to Meet, Epistemic Norms: What is the Motivation?” describes a (non-exhaustive) range of specific circumstances in which epistemic goals may co-operate or compete in various ways with motivational goals, and suggests specific avenues for future research. The Section entitled “Motivated Explanation from a Cognitive Neuroscience Perspective” reviews work on the neural implementation of core explanatory processes, of motivation and reward in general, and of possible pathways for interaction, and makes further recommendations for future work. Finally, the Conclusion summarizes our review and proposed framework for motivated explanation.

**Table 1:** **Component processes in the proposed framework for explanatory reasoning**.

**Phase of explanation**	**Mental process**	**Description**
Generating explanations	Activation	Intuitive judgment on criteria for what qualifies as explanatory
	Memory search	Episodic and semantic memory retrieval of prior events, explanations, or statistical patterns relevant to the target of explanation
	Cognitive updating	Integration of new information and prior knowledge; can involve reinterpretation of information in memory
Evaluating explanations	Coherence judgment	Evaluate “fit” with prior knowledge; can also judge coherence of explanation with a particular psychological state
	Weighing evidence	Assign value to evidence to compare it against other evidence, or some predefined threshold
	Simplicity judgment	Evaluate number of assumptions or causal mechanisms involved in an explanation, and the joint probability of their all being involved
	Credibility judgment	Intuitive judgment of plausibility; use when other criteria are ambiguous, or when explanations compete
	Breadth judgment	Judge explanatory flexibility to account for multiple events/concepts across contexts
	Depth judgment	Judge whether the explanation accounts for the details of the event or concept being explained

## Explanatory processes

### Generating explanations

We propose that explanations originate from three generative processes (see also Lombrozo, 2006):

- **Activation** of a general sense of what is explanatory, or what is essential to being an explanation. This includes our judgment of when some factor is “the real explanation (or cause)” rather than just a background condition. It also covers such questions as whether an explanatory connection is a necessary one (e.g., does a cause necessitate its effect?); the expected temporal order of causal relations; whether there can be true “action at a distance”; and whether explanatory connections essentially involve fitting explainer and explained into a larger, recognized pattern. There are no definite, agreed upon answers to such questions, and even everyday intuitions are subject to change with circumstances. However, for our purposes it is not necessary to propose definite answers. What we suggest is simply that people do at least implicitly consult their intuitions about what counts as a cause or explanation, and that these intuitions act as an early-stage filter on the overall process of generating candidate explanations.
- **Memory Search** for candidate “off the shelf” explanations, and for potentially relevant events or associations of various sorts, is a major part of the construction process. Note that one can re-do a memory search at some latter point as one generates explanations, casting a wider net or focusing the search more narrowly if initial efforts do not produce anything adequate. It is important that not only are our memories subject to all the familiar sorts of manipulation at encoding, during “storage,” and at retrieval, but also that they are subject to *re*interpretation in light of current goals, including explanatory goals.
- **Cognitive Updating**. Normally studied in laboratory settings as the ability to change or add to representations being held in working memory, cognitive updating covers many sorts of manipulation of information (e.g., searching for new pro or con considerations, reinterpretation of old memories, “on the fly” construction of explanatory hypotheses, (re)assigning weights to relevant factors, (re)evaluation of thresholds levels of credibility for candidate explanations, judgments of coherence with background knowledge). These are all subject to motivational influence both epistemic and directional.

### Evaluation processes

We suggest that six processes enter into the evaluation of explanations, although not all need to be involved in every explanation. Some of these evaluative processes appear in the construction process as well, due to the fact that construction and evaluation are often not temporally distinct, but overlapping processes. For example, we may evaluate explanations as they are emerging and abandon the construction process if a candidate starts to look implausible. Or we may intuitively evaluate some bits of evidence or testimony as especially important even if we do not yet know why or how, and thus work to include them as we construct candidate explanations.

- **Judging Coherence** or “fit” of a potential explanation with background assumptions. Conceptual coherence relies on appeals to unifying theories or causal models (Murphy and Medin, 1985), and so can be sharpened to the extent that such theories or models are made explicit. One can also speak of emotional coherence (Thagard, 2002), and we will see how this might be distinguished and prove helpful in connection with specific cases considered in the Section entitled “Competing Directional Motivation.”
- **Weighing of Evidence**. This is often intuitive, since there are seldom explicit criteria for what evidence is important. What might seem to be a minor detail can turn out to have major importance (a telltale “clue” spotted by Sherlock Holmes, or the precession of the perihelion of Mercury). Note also that weighing of evidence interacts with coherence judgments, in that some discrepancies may seem to involve only minor points, while others appear more important, so that the former will seem to constitute important evidence, but not the latter. The weighing of evidence for or against candidate explanations is as important in evaluating explanations as the weighing of attribute importance is in determining category membership, and is an important point of entry for the influence of directional motivation.
- **Judging Simplicity**. Parsimonious explanations are often preferred to those requiring more assumptions and other prerequisites, but it may be that people do not favor (on epistemic grounds) the simpler explanation even if other factors appear equal (Johnson-Laird et al., 2004). This depends in part on how one defines or measures simplicity. Nonetheless simplicity judgments are important in many explanatory contexts, intuitive as they may be (Lombrozo, 2007). Note that there is sometimes a “trade off” between coherence and simplicity, for in principle the simpler of two explanations is not necessarily the same as that which coheres better with background beliefs.
- **Judging Credibility** of a potential explanation. This is not an entirely independent factor, since coherence can be a large part of it, as can simplicity (For example, a candidate explanation that fails to cohere in major ways with our background beliefs will seem implausible to us; so also for explanations that appear wildly and unnecessarily complicated). Nonetheless there is a general recognition that at least an intuitive judgment of credibility enters into our evaluations when other criteria are not decisive. The largely intuitive nature of credibility judgments leaves them especially vulnerable to directional bias.
- **Judgment of Breadth**. A simple measure of comparative breadth is that one explanation (E1) is broader than another (E2) if E1 explains everything E2 explains and explains other things as well. In particular cases the comparison may not be straightforward, but often it is. For example, Newton's laws of motion were by that measure clearly broader than Kepler's laws of planetary motion or Galileo's laws of terrestrial motion.
- **Judgment of Depth**. We note three familiar conceptions or uses of “depth.” E1 is deeper than E2 just in case:
  - E1 is fuller or more detailed, than E2, or
  - E1 includes more fundamental level(s) of explanation than E2. For example, there are many levels of explanation for psychological phenomena, from that of historical trends and social forces down through individual psychological processes, then to neural implementation, and so on through levels of physical analysis. Sometimes more fundamental explanations are also broader, as when Newton's laws postulated a very fundamental force of nature (gravity) to explain everything explained by Kepler's and Galileo's laws of motion, and much else besides.
  - E1 is given in more abstract/general terms than E2, pushing toward “virtually mathematical” terms (Strevens, 2008). This sort of depth is especially conspicuous in modern theoretical physics, but also in Ancient theories of musical harmonies and celestial motions in terms of mathematical modeling.

When multiple evaluative considerations are involved they may not unanimously favor the same explanation. Thus, there will again be a problem about how different factors are to be weighted. There is relatively little discussion of this problem at present, and no solution—which is to say that although relative weighting may sometimes be obvious and formulaic, it is often in practice very much an *ad hoc* and flexible judgment. As with intuitive credibility ratings, this opens wide the door to directed motivational influences.

Each of these generative and evaluative processes constitutes a point of possible entry for motivational influence. More general studies of motivated reasoning have as a rule focused on one or another specific consideration, but some researchers have suggested rules of thumb for such situations: while arriving at a desired directional explanation people attempt to maintain an “illusion of objectivity” (Kruglanski, 1980; Pyszczynski and Greenberg, 1987); or, they draw the desired conclusion only if they can muster reasonable evidence for it (Darley and Gross, 1983); or they try to construct a supporting case “that would persuade a dispassionate observer” (Kunda, 1990). On these views our directionally motivated explanations are either constrained by the usual standards of accuracy (“mustering reasonable evidence,” “persuading a dispassionate observer”) or at least by the appearance of meeting those standards (“illusion of objectivity”). The last of these—creating the illusion of accuracy—would of course apply to cases of deliberate deception as well as to unwitting self-deception. Within those constraints people may engage in biased processing of various sorts in order to move things in the direction of a desired explanation. This general picture works well up to a point. However, some of the cases we survey below involve witting or unwitting departures even from those rather flexible epistemic constraints.

We turn now to discussion of concrete cases of motivated explanation and of how motivation interacts with the processes just surveyed. All of these make clear that despite the implication of the phrase, “*the* best explanation,” there is seldom if ever a uniquely “best” explanation. This is obvious in the sense that there may be one epistemically best explanation of a specific type (e.g., mechanical, teleological), and at a specific level of analysis (psychological, neural), but other epistemically best explanations for other levels of analysis. More importantly for present purposes, we often have directional motives in addition to, or instead of, the motivation to achieve accuracy at a certain level. The best explanation for those purposes may not be the same as that which best meets epistemic norms.

## To meet, or not to meet, epistemic norms: what is the motivation?

### Directionally enhanced epistemic motivation

Motivating people to be accurate—for example by telling participants that their results will be judged by others, or made public, or will affect the lives of others—can produce more extensive and careful processing, thus reducing some cognitive biases (Kassin and Hochreichl, 1977; Kruglanski and Freund, 1983; Tetlock, 1983, 1985; Harkness et al., 1985; Tetlock and Kim, 1987). Add to this the natural motivation to reach an accurate explanation whenever that is critical to one's own well-being, and one can appreciate that humans often attempt to meet epistemic norms, with directional motivation enhancing epistemic motivation.

### Competing directional motivation

Still, humans have a strong tendency to accept and give to others the explanation that best suits their purposes in a particular situation, and these often call for the relaxation or outright violation of epistemic norms. Directional purposes may override epistemic motivation, or may be overridden by it; or the two might both contribute to a compromise solution. We turn now to a series of cases illustrating the importance of explanatory motivation and pointing the way to future research.

#### Confirmation bias

Confirmation bias is the tendency to gather, remember, or interpret information in a way that confirms one's own views (Tversky and Kahneman, 1974). An even broader confirmation bias can be found in connection with hypotheses under consideration and to which one does not yet have any particular commitment. We suggest four specific ways in which a general explanatory confirmation bias might be implemented. First, people might interpret a question about the truth or accuracy of some explanation as the question of whether there are any good reasons to believe it. This would trigger a search for such reasons—i.e., for considerations that confirm the explanation. Second, even critically-minded people might prioritize their responses, looking first to see whether the view is defensible, then for evidence that it is false. To the extent that the first search turns up what appears to be convincing positive evidence, this could undermine a subsequent search for negative considerations, for as one became increasingly firmly convinced of the truth of some theory or explanation, one's willingness to invest in a thorough investigation of negative considerations could weaken. From a cost-benefit point of view, further investigation might come to appear not worth the time and effort (Beach and Mitchell, 1978; Payne et al., 1988). Thus, even if one initially “has an open mind,” the tendency to look first for confirmation might, if successful, interfere with an initial intention to look without prejudice at both sides of the question. Third, and by contrast, if a confirmation search turns up little or nothing in the way of support for a possible explanation, one might not think it necessary or worthwhile to look for disconfirming evidence. One's overall motivation will determine whether or not there would be sufficient point in pursuing the matter further.

A fourth, related, consideration is that the discovery of convincing positive confirmation can generate an excess of enthusiasm for and confidence in a given theory, which may truncate the search for alternatives and bias the evaluation of rival theories. For example, in cognitive science one sees the accusation (Churchland, 2011) lodged against some evolutionary psychologists that they tend to spin out and give credence to evolutionary “just so stories” while neglecting other potential explanations, because those support their own postulation of a multitude of “modules” that have evolved to solve specific sorts of problems. There is nothing disingenuous in this, of course. It is rather a matter of growing confidence in and enthusiasm for a theory biasing one's search for and evaluation of candidate explanations (We take no stand here on whether the accusation is justified in this case).

All four factors can support a general confirmation bias. We suggest, however, that it is in cases of specifically “my-side” bias (i.e., where one already has a commitment to or a preference for some potential explanation) that one finds the influence of distinctively directional motivation. There is no one sort of motivation in my-side bias, but an open-ended array of potential sorts of motivation—maintaining self-esteem, shifting blame, reconciling social conflict, etc. These thus become special cases of the sorts of directional motivation we consider in the following sections.

#### Resolution of cognitive dissonance

The study of cognitive dissonance (the negative affect accompanying perceived conflicts between our beliefs and new information, or between our values and our statements or actions), is 60 years old and going strong (Festinger, 1962; Harmon-Jones, 2004). When such conflicts occur, the negative affect they produce motivates one to resolve the conflict. It now appears that actual arousal, including measurable physical arousal, is crucial to dissonance reduction (Cooper et al., 1978). Similarly, the agent must attribute the arousal to some aspect of his own freely chosen behavior, thus taking responsibility for the negative outcome that is the source of the arousal, rather than attributing arousal to some external source (Zanna and Cooper, 1974; Cooper and Fazio, 1984). Resolution may take different forms: changing our behavior, beliefs, values, attitudes, or some combination of these. Interestingly, in cases involving self-esteem (e.g., acting contrary to one's own standards of what is right), people do not engage in belief or attitude adjustment if some alternative means of boosting self-esteem is ready at hand (Steele and Liu, 1983). Where attitude adjustment does take place it now seems clear that people do not normally shift their beliefs or attitudes freely, but under some constraint from prior beliefs. Thus, adjustments are in a desired direction, but represent a kind of compromise outcome (Kunda, 1990) or balancing of epistemic and directional motivations.

There is a direct link between dissonance reduction and motivated explanation in that the latter is a commonly used means of resolving the former. When conflict arises between what we do and what we believe, for example, we often attempt to resolve the resulting dissonance by bringing our beliefs about why we did what we did into harmony with our actions or with one another. Children's explanations of their own behavior (e.g., refraining from playing with a fun toy) can be influenced by manipulations as simple as the difference between prospectively mild and severe punishments (Carlsmith and Aronson, 1963). When facing the threat of severe punishment, children cited the threat of punishment as the reason they refrained from playing with a fun toy, whereas with only a mild threat, children tended to say they refrained because the toy “isn't very fun” — a clear case of “attitude adjustment” to remove conflict between attitude and behavior. A broad range of cases have been studied behaviorally among adults, including “counter-attitudinal” essay writing or speech giving, and “induced conformity” studies (Cooper, 2007). Connectionist models and neuroimaging studies of cognitive dissonance have also appeared (van Veen et al., 2009).

These behavioral studies all recognize some form of directionally motivated explanation, and all recognize the need to study the effects of manipulating affect or emotion. We suggest that non-epistemic motivations of various kinds influence the vast majority of our explanatory activities, and that one task for future study of explanation is to design behavioral and imaging experiments so as to manipulate in a controlled way the presence of specific sorts of affect or emotion in explanatory contexts, and to determine how this presence makes a difference to specific explanatory processes. Work on dissonance reduction has achieved this to some extent, but the range of motivational influences is far greater than has been studied so far. Further work will require a combination of context manipulation, self-reporting (on emotional experience and on explanatory procedures) and imaging evidence.

We also suggest that longitudinal studies be undertaken to determine whether, or to what extent, attitude shifts involved in dissonance reduction are merely temporary. With the passage of time, arousal will usually abate, and may not recur as one thinks back on the original dissonance-producing behavior; in other cases, recollection of past action may continue to constitute painful (embarrassing, shameful, guilty) memories. Is the latter situation one in which our attitude shift is convincing (to us) and lasting? In the former situation do we maintain our attitude adjustment only temporarily, so that now we are able to acknowledge that what we did really was foolish or bad, and to recognize that our attitude adjustment was our way of avoiding such acknowledgment? One could extend behavior attribution and induced conformity experiments to assess whether the attitudinal changes persist in time and how their temporal history relates to the presence or absence of dissonance.

#### Reasoning from inconsistency to consistency

Reasoning from inconsistency to consistency in belief is akin to cognitive dissonance reduction, but need not always involve actual dissonance. A relatively recent study (Johnson-Laird et al., 2004) of inconsistency resolution does not address cognitive dissonance, focusing instead on the process of reasoning itself rather than the nature of the motivation behind it. The authors correctly stress the frequent role of explanatory thinking in bringing our beliefs into consistency and emphasize the need for further work on how we generate explanations. Their interest is in how people construct mental models reflecting simple deductive explanations such as: If Paolo went to get the car, he will be back in 5 min; Paolo went to get the car; Therefore, Paolo will be back in 5 min. When Paolo fails to return in 5 min there is a contradiction between this new fact and the conclusion of one's previous deduction. Restoring consistency involves a series of three processes: detection of the inconsistency; withdrawal of (at least) one of the premises of the initial deduction; generation of an explanation for Paolo's failure to reappear. The authors then describe how people carry out those processes in terms of either complete or incomplete mental models of possibilities representing the relevant propositions and the logical relations among them. Depending on whether or not people construct complete or incomplete mental models, the theory predicts that in generating an explanation so as to remove the inconsistency they will tend to reject the categorical premise or the conditional premise of the initial reasoning, respectively. Somewhat different predictions hold if the first premise is a biconditional. Experimental results support these predictions.

We have commented elsewhere on what we take to be the virtues and the limitations of this specific approach to causal thinking in terms of mental models (Patterson and Barbey, 2012). Here we suggest that the aim of removing inconsistency by finding the most probable explanation is only one motive (the “accuracy” or “epistemic” motive) at work, and that other motives can heavily influence the specific manner in which we arrive at an explanation that removes the contradiction, and can exert this influence at multiple stages in the generation, evaluation and selection of a “best” explanation.

For example, we agree that there are numerous ways one might explain Paolo's non-reappearance: he cannot find the car; he has made a wrong turn on the way back; he is stuck in traffic; he has he run off to Buenos Aires with his secretary, etc. (Johnson-Laird et al., 2004; we supplement slightly their stock of possible explanations, with apologies to Paolo). All of these would explain the situation and resolve the contradiction. There are potentially many different explanations that involve denying the conditional premise of the simple deduction cited above, and numerous others that would entail denying the other premise. The issue of interest to us, however, is how motivation affects the generation of specific explanations of Paolo's lateness, and these effects are not accessed via this experimental paradigm. For the moment we simply stress the potential importance of directional motivation in constructing such explanations, regardless of which of the premises are eventually rejected—and, it is important to add, regardless of whether one frames the initial situation in terms of a deduction, as in Johnson-Laird et al. (2004). Thus, in the next section we use the Paolo example to explore the possible influence of directional and especially emotion-driven motivation on how one explains Paolo's tardiness.

#### Emotion confirmation, emotion regulation, and emotion-driven explanation

We suggest that there is an “emotional confirmation bias” analogous to the familiar cognitive confirmation bias, in that we are typically motivated to favor explanations and beliefs that confirm our emotional response to some agent, event, or situation as regards its nature, intensity, and duration. Dissonance reduction studies assume at least implicitly that dissonance creates, or itself constitutes, motivation for its own down-regulation. But precisely how we down-regulate (by modifying our beliefs, behavior, or values) is another question, and here emotion, and any affect present in addition to cognitive dissonance, can have a decisive effect. For example in the Paolo case his lateness might anger us so that we are receptive to explanations that not only remove the inconsistency and/or cognitive dissonance, but also justify our anger (“He's probably just taking his sweet time, even though it inconveniences several others”). Our proposal is that there is a general motivation (and bias) toward confirmation of one's emotional or affective state, where this may happen to produce down-regulation, or up-regulation, or neither. Thus, emotional confirmation bias is very wide-ranging, as is the analogous cognitive confirmation bias.

Although we have a general motive to justify our emotions, and although this will often issue in attempts to explain them in a way that shows them to be “reasonable” or appropriate, it is also true that while in the grip of strong emotion such as rage, jealousy, or hatred we sometimes justify or rationalize our response by devising explanations that seem, at least to less involved or dispassionate observers, to be rather arbitrary or even quite irrational. This suggests a modification of Kunda's proposal that directionally biased explanation is constrained by the need to arrive at an explanation that would be considered plausible by a dispassionate observer (Kunda, 1990). That is very often true, but powerful emotion can override even that degree of constraint. As a corollary we suspect further that in this type of situation an extreme bias in explanation will have a temporal history roughly parallel to that of the strong emotion driving it: if over time the emotion fades, one may retreat to a more epistemically respectable explanation, admitting for example that one had angrily “over reacted,” and perhaps proposing an explanation for why one over reacted.

Returning to the Paolo example, where strong emotions enter the picture we have equally strong motivation to arrive at explanations that not only remove cognitive dissonance or any logical contradiction arising from his surprising lateness, but to do so in a way that confirms our feeling toward him. We also call more readily to mind and weigh more heavily background information that supports such an explanation. Again, emotional confirmation bias motivates us to generate and accept *some* explanation of our emotion that supports its appropriateness, and it can influence our thinking via more than one explanatory process (here, for example, comparative credibility ratings on candidate explanations, memory search for explanations and for evidence, and weighing of evidence).

Future research on the influence of emotional factors in explanation will need to vary emotional motivation while holding other factors in the explanatory situation constant. This would be parallel to work aimed at varying epistemic motives, as by telling participants that their explanations would be evaluated by others, or in general that they would be held responsible for the accuracy of their explanations. Although anecdotal evidence for an emotional confirmation bias is all too easily found, systematic investigation will require manipulation of a variety of emotions in a controlled, measureable, and ethically permissible manner, and this will be easier said than done. That is not to say that it cannot be done, although there will be no more simulated electric shock or prison experiments like those of yesteryear. Appropriate story materials or videos might prove a useful if limited instrument (Soussignan, 2002).

Finally, research should look not only for the presence of emotional confirmation bias, but also for how emotion influences various specific processes involved in the generation and evaluation of explanations. We expect that judgments of simplicity, breadth and depth will be *relatively* resistant to such influence, but that many other processes (e.g., memory search, weighing of evidence, judgments of credibility) will be highly susceptible, even if not quite “pliant as a windblown sleeve.”

#### Rationalization of judgments and actions

Rationalization, in the sense of explaining one's judgments or behavior so as to put them on a “rational basis” or, when there is some question about their propriety, to justify them, produces familiar instances of motivational interaction and interference with epistemic norms. We focus here on one type of case currently under especially active investigation today—that of rationalizing one's moral judgments—then indicate how our proposal applies also to a broader range of cases.

There is good evidence that people tend to think that they can explain their responses to questions about moral choices by the reasons they have for responding that way. But there is also evidence that for the most part our moral judgments are in fact quick and intuitive, and that the justifying explanations we give for them are usually *ad hoc* and do not reflect the actual basis of our judgments (Haidt, 2001). Even when, in the course of a moral interview all of one's explanations are shown to be inadequate, so that one becomes aware of the fact that one has no viable reason for some particular judgment, one often continues to affirm the judgment (e.g., “I just know it's wrong, that's all”). This is a familiar result, from Socrates' cross-examinations of fellow Greeks to the recent experimental work of “social intuitionists” (Haidt, 2001; Greene et al., 2004). We have argued elsewhere (Patterson et al., 2012) that this social intuitionist account of moral judgment leaves out a large if indirect role for reasoning, especially on the part of one's early cultural influences such as teachers, family, legislators, etc., in the creation of our moral intuitions themselves, hence an indirect role for reasons and reasoning in the generation of our intuitive moral judgments. Nonetheless, the evidence does strongly suggest that people are routinely motivated to explain or justify themselves; that they assume their rational justifications (if any) explain their judgments; and that their judgments are often not in fact based on any reasons that they themselves are able to supply.

From our point of view the important facts are first, the presence of a motivation to give one's moral judgments or behavior a rational or justificatory basis, especially if one has been called into question, and second, the potential conflict between the motivation to justify oneself and the goal of reaching the best explanation as defined by epistemic norms. Epistemic and self-justifying motives sometimes join forces rather than compete—as with the innocent but wrongly accused person trying to prove her innocence—and this can produce an especially thorough search for evidence, careful weighing of evidence, and so on. But sometimes the explanation that best satisfies epistemic norms is not the same as that which best suits other purposes. When the two diverge we may or may not be aware of this; thus we may either sincerely believe the two explanations coincide when in fact they do not, or we may be aware that they do not coincide, but cynically protest that they do. In the latter case we typically try to make it appear that the epistemic motive has been served even if we know very well it has not.

Richard Nixon, for example, may have sincerely believed that the suppression of certain information about Watergate (the “cover up”) was motivated by “national security” concerns or, later on, by concern for “the Presidency itself.” To many observers a better explanation seemed to be concern for “Nixon security” or “this President himself.” Some of the latter observers also thought that Nixon himself was well aware of this self-serving motivation, and that the true explanatory motive (survival as President) drove the formulation of the nobler, self-justifying, explanation deceitfully offered to the public and to Congress. But it is possible that Nixon himself believed in some high-minded—and just coincidentally, Nixon-justifying—explanation of his actions. Whatever Nixon or his supporters actually believed, his directional motives would have disposed him to recall, cite, and give special weight to the sorts of background information that would support self-justifying explanations. Thus, supporters would cite, and consider compelling, evidence on one side (e.g., “He has always proved a staunch defender of national interests, all the way back to rooting out dangerous Communist spies and sympathizers in the 1950's”). By the same token, detractors would think of, and find compelling, any evidence for the opposite conclusion (e.g., “He has always been cynical and self-serving, all the way back to his days as a Red-baiting Congressman in the 1950's”).

Thus, each side tends to interpret past actions so that they fit into a recognized pattern that in turn supports a desired conclusion. This fitting of events into a pattern is an important feature of many explanations (Friedman, 1974), and is an important aspect of establishing the coherence of an explanation with background information. But the striking aspect of the Nixon situation was that the two sides, under the influence of two very different directional motivations, interpreted very differently many of the “same” actions spanning many years of Nixon's career, thus producing two very different patterns of behavior—one self-serving and opportunistic, the other admirably civic-minded.

In both cases the directional motive tends to influence explanation in a number of ways: by influencing memory search for relevant information, weighting of the importance of pieces of evidence, interpretation of past behavior, failure to consider seriously alternative interpretations, and failure to recognize the ambiguity of one's own evidence. In the end each side's explanation not only appears to its adherents to satisfy epistemic norms, but also appears to them to confirm the prior beliefs about Nixon that helped produce those explanations. In light of the importance of emotional factors in this case we suggest also that both sides' explanations served the directional motive of justifying their adherents' strong emotional attitudes toward Nixon as a person or his policies.

The interplay of various motives in this case illustrates the manner in which our explanatory thinking may involve multiple interacting motivations, of which the purpose of meeting epistemic norms is only one. In “real life” and especially in personal or social situations, there will often be multiple and conflicting motives involved. Explaining how these jointly influence our thinking as we arrive at a kind of maximally satisfactory explanation in a particular situation is one of the great challenges for the study of explanatory thinking. Where strong emotion is involved, dispassionate assessment of the pros and cons of possible explanations requires a major effort and good deal of self-discipline.

Finally, the Nixon case illustrates clearly the influence of directional explanatory motivation on the interpretation and possible *re*-interpretation of information that one does recall and consider significant. We noted briefly just above how some of Nixon's past actions were interpreted in radically different ways by friends and foes. But events can also be re-interpreted if one revises one's estimate of some agent. If the weight of evidence brings some observers to change their estimation of Nixon's character, this will probably result in re-interpretation of some aspects of the man's past career. Once more, we suggest that this is especially likely when strong emotion is involved, whether in cases of national politics or private affairs. Thus, although studies of motivated thinking rightly emphasize the importance of memory search, it is important to note further that there is not a fixed range of past experience through which one then searches in either a biased or unbiased manner. Rather, to a significant extent the past is subject to interpretation and reinterpretation in light of our current explanatory motives. Events that did not seem significant before may now appear crucial for explaining something important. Or, under changed circumstances (e.g., if the accumulation of evidence persuades us that Nixon is not, after all, “a crook,” as he put it), then we may revise our past interpretation of—and explanations of—Nixon's actions. This interpretation and reinterpretation of the past can occur not just as we attempt to explain the actions of others, but also as we try to understand our own actions or judgments.

#### Avoiding responsibility, shifting blame, making excuses

Cases of avoiding responsibility, shifting blame, or making excuses overlap with those of self-justifying rationalization, and of dissonance resolution as well; even so, they constitute special cases deserving notice in their own right, in that they almost invariably involve specifically explanatory motivated thinking. Where one's behavior is admittedly questionable or wrong, one may try to avoid responsibility: “The situation left me no choice”; “Under the circumstances I felt I had to do it, even though I didn't want to.” In a legal setting one might explain one's action as a “crime of passion,” or as due to “temporary insanity.” In more mundane settings, we may attempt to lesson our responsibility with the excuse, “I just don't know what got into me”; “Sorry; I'm just very stressed today.” In these last cases the causal explanation (excuse) suggests either that it wasn't the “real me” who gave offense (it was something that got into me; it was just “John Barleycorn talking”), or that I did not really choose the action, but was compelled (by overwhelming passion, or a situation permitting no other course of action), or at least that there were “extenuating circumstances” (stress, bad hair day) that partially explain one's action. Such excuses can certainly be disingenuous, but we sometimes sincerely attempt to explain ourselves to ourselves or to others by finding an explanation that (a) seems to us, in our circumstances and state of mind, credible and (b) serves to lessen our responsibility. We often find particular explanations plausible, and better than other explanations, at least in part because they fulfill our non-epistemic, self-serving, purposes. Again, we do not suggest that there is no limit on what we can believe about ourselves; rather, directional goals can shift our explanations in a self-serving direction. But by the same token we suggest once again that this effect will be more pronounced when our non-epistemic motives include powerful emotional motivation.

For reasons we need not belabor, similar remarks apply not only to making excuses for ourselves or justifying our own behavior, but also to shifting blame from ourselves onto someone or something else. This begins in early childhood with the simple and multi-purpose “He started it,” and continues, with variations, into adulthood and even onto the international stage—as with long-term hostilities in which both sides explain and justify their own actions by reference to some previous action on the part of their adversary.

#### Systematic self-deception: our rose-tinted glasses

Every type of directionally motivated explanation potentially involves self-deception, and we have already noted several examples of this. We add here several types familiar from studies of other phenomena in order to bring out their dependence on motivated explanation in particular. One type involves biased general assessments concerning oneself, as well as local bias concerning a particular situation. For example, most people are unrealistically optimistic about their chances in life, their “potential” (Sharot, 2011), their influence, the impression they create on others, their own virtue, the robustness of their health, or as Socrates remarked, their own intelligence or good looks (Plato, *Philebus*, c. 355 BCE, in Cooper and Hutchinson, 1997). For example, we are inclined to attribute a rival's success to “luck,” or to regard our team's failure as a “fluke.” People systematically over-estimate the causal roles of others' personal traits in determining their actions while underestimating the influence of situational factors, a phenomenon known as Fundamental Attribution Error (Ross, 1977). We tend to rate the reliability of a medical diagnosis higher or lower, depending on whether we want to believe the diagnosis (Jemmott et al., 1986; Ditto et al., 1988), so that we implicitly suggest a “false positive” explanation for a bad test result); we find more (or less) convincing a study emphasizing the risk of caffeine for women, depending on what the study implies about our own risk (Kunda, 1987). These cases can be psychologically complex, involving confirmation bias, dissonance reduction, and other factors. What we emphasize here is that in some cases the recognized tendency to look at things through “rose-tinted spectacles” also systematically influences our explanatory thinking.

Fewer people are unrealistically negative in their general outlook (e.g., pessimists and hypochondriacs), but bias toward either the positive or negative typically involves a failure to rigorously apply epistemic norms to explanatory thinking. There is considerable evidence that this can be beneficial to one's mental health, at least in people who manage to maintain an optimistic outlook, so long as they do not disastrously underestimate certain risks (e.g., by dismissing the importance of an unwelcome medical test result). Thus, there is a very widely-shared even if implicit motivation at work in much of our explanatory thinking about ourselves, others, and the world in general. Indeed, biased explanatory thinking in particular cases will tend to reinforce and perpetuate a “rosy” outlook, which will in turn influence further particular overly-optimistic explanations. If this sort of thing is, within limits, beneficial, it is in that respect a good thing, and even in one clear sense rational to depart from strict adherence to epistemic norms. Given also that epistemic rigor can be difficult to achieve, using resources of time, energy, etc., it is all the more reasonable to forego setting the epistemic hounds loose on our optimistic interpretations and explanations of ourselves—again, unless special circumstances indicate that greater “realism” is needed. Since it also appears that we are almost constantly weaving explanatory narratives about ourselves (Lombrozo, 2006), it is fair to say that a very large number of our self-explanations are biased by the purpose of maintaining a view of ourselves and our world that is in fact unrealistically positive—or in some people, the opposite.

This is related to the familiar and more general fact that when it comes to self-understanding, most people tend to trust their own explanations farther than the evidence warrants (Ostrom and Walker, 2003). We suggest that generating at least credible self-explanations not only nudges us toward belief in those individual explanations, but also strengthens a positive general assessment of ourselves as cognitive agents.

#### Giving explanations: pedagogy

Giving explanations can involve further processes beyond those involved in generation and evaluation of explanations for accuracy. In a broad sense one can always regard the latter processes as part of giving an explanation to oneself or to others, so that all the explanations surveyed so far have been produced in order that they might be given to someone to serve some purpose or other. But teaching—via lectures, textbooks, private instruction, etc.—highlights explicitly some important explanatory challenges that are either left implicit or are simply not relevant in many explanatory contexts. In rough terms, let us think of teaching as in part the giving of explanations by someone more expert to someone less expert. Here giving the most accurate explanation known to the teacher may not work, if only because the learner is not yet equipped to understand it. So a less comprehensive, simplified, analogical, metaphorical, diagrammatic, or pictorial explanation is called for. As the learner progresses, explanations can become fuller, deeper, and closer to an epistemically best explanation of a particular type. Here the overarching motivation is pedagogical—to help the learner advance in understanding—but is broken down into a series of progressive educational stages with corresponding levels of explanation.

Outside “official” educational contexts one meets similar explanatory challenges. These we typically try to meet on an ad hoc basis, again attempting to assess the explainee's current state of comprehension, cognitive resources, level of interest (which we may try to up-regulate), and any practical ends to which the explanation will be put, in order to generate an explanation that will advance understanding toward the level of sophistication and accuracy called for by a specific situation. As always, we may try to deceive someone; and even if not, we may deliberately offer a less-than-epistemically-optimal explanation in order to get across what is truly important for the purpose at hand (e.g., Feynman's explanation to Congress of how the Challenger exploded).

Here the study of motivated explanation intersects with a large body of empirical—and sometimes controversial—work in education studies. We do not undertake a survey, much less an independent evaluation, of work in the latter field, but simply stress the sometimes critically important influence of a pedagogical motivation on our explanatory practices along with the special challenges it brings—hence the possibility for future work to take advantage of existing results about what works and how in pedagogical contexts.

## Motivated explanation from a cognitive neuroscience perspective

We now turn to an examination of the neural mechanisms that underlie the observed role of motivation in human thought and explanation. We briefly survey two areas: research on motivation and reward systems, and research on the neural substrates of motivated reasoning. Specifically, we review how the processes of thought substitution and thought inhibition contribute to motivated explanatory reasoning by influencing the generation and evaluation of explanations.

### The neural correlates of motivation

The cognitive neuroscience of motivation in humans has revealed a cortical-subcortical system supporting representations of reward and value that are used to modify behavior in pursuit of goals. Human goals include that of finding epistemically good explanations, in addition to finding explanations that serve a wide variety of directional goals. Motivation as the force behind goal-directed behavior (as opposed to reflexive and automatic observed actions) is thus supported by a cortical-subcortical system linking hedonic experience to action representations. Subcortical structures including the striatum, ventral tegmentum and nucleus accumbens form a network using dopamine to signal the receipt and anticipation of reward (Elliott et al., 2000; Samejima et al., 2005). Note that the same dopaminergic circuitry supports procedural memory and conditioned learning, as part of a more general, distributed brain function of calculating and minimizing prediction error (Berridge, 2007; Chiu et al., 2008; Rolls et al., 2008). Cortically, subdivisions of the frontal lobe represent and manipulate information about rewarding stimuli, among the other diverse sources of information to which the frontal lobes are sensitive. The ventromedial prefrontal cortex (vmPFC) is believed to support reward-oriented and risk-averse behavior by representing signals from the striatum when paired with visual or other predictive signals (Rolls, 2000; Kringelbach, 2005). Together with the anterior cingulate gyrus, activity in the vmPFC also enables the updating of reward-related paired associations when environmental contingencies change (Rolls, 2000; Hornak et al., 2004; Hampshire et al., 2012). vmPFC activity scales proportionately with the subjective value of rewarding experiences, while dorsolateral activity modulates the intensity of reward representation according to the context of goals and intent (i.e., when a particular stimulus is no longer “rewarding” according to a person's changing goals, the dlPFC supports the attenuation of the stimulus-reward mapping; Hare et al., 2009, 2011).

It is controversial whether motivation is supported directly by the neural correlates of hedonic experience, or instead by predictive signals and associative learning systems alone, but hybrid views account for both (see Volkow et al., 2002; Berridge, 2007, for competing theories, and Ettenberg, 2009, for the hybrid view). What the theories have in common is that regardless of the view on motivation and reward having dissociable neural correlates, motivation clearly relies on linking prior rewards to the prediction and pursuit of future reward.

This is important to the discussion on motivated reasoning, because at its core, the existence of motivated reasoning relies on the ability of an individual to predict emotion states that are linked to a particular idea being true (or, as in several cases discussed above, to an idea's appearing to be true to some relevant agent), and then adjust the reasoning process accordingly. This applies to any of the examples of non-epistemic motivations discussed here: self-justifying bias, confirmation bias, dissonance resolution, blame shifting, escapism, and so on. By analogy to goal-directed behavior, in which we use input from the reward-motivation system to predict action outcomes and adjust behavior accordingly, there must be similar input from the motivation system in reasoning under non-epistemic motivations that assigns value to a given explanation, based on its predicted association with some directional goal.

As others have noted elsewhere, the exact mechanisms linking reward representation, motivation and action control processes in the brain have yet to be fully explored. Exploring the similarly ambiguous link between motivation and reflective reasoning processes is likely to shed light on the motivated decision-making process as well.

### The neural correlates of motivated reasoning

Relatively few studies have directly measured the neural basis of reasoning under varying motivational states. The realm of political beliefs is one exception, and one in which people are measurably susceptible to the forces of non-epistemic motivations. Presented with some discrepancy between a politician's statements and actions, we might be more motivated to look for circumstances beyond the politician's control or to blame the politician's character flaws, depending on our party affiliations. Self-described “committed partisans” (e.g., those involved in the Nixon case discussed previously) in an fMRI study were shown discrepancies between the statements and actions of political leaders in their own parties, to compare the neural response measured to that when considering the actions of neutral candidates (Westen et al., 2006). The posterior cingulate and precuneus were activated by observing any conflict between statements and actions, regardless of the politician's party affiliation. The desire to explain a person's actions favorably in spite of conflicting evidence also engaged the vmPFC and ventral anterior cingulate gyrus: regions generally implicated in emotion processing and reasoning. When compared with later consideration of exculpatory evidence, the initial consideration of contradictory evidence engaged the left lateral inferior prefrontal cortex and left insula, which are both implicated in interoception, self-concept and negative emotion. The effect of political party affiliation in this study paradigm indicated that attention and conflict monitoring systems are engaged when people come across inconsistencies, but ventral emotion-processing systems come online when a person's search for an explanation is also constrained by the desire to draw a conclusion maintaining favorable self-image or consistency of beliefs. The brain processes information differently depending on the consequences that the information at hand has for our desired conclusions or end states.

Returning to the other non-epistemic motivations that could influence a reasoning process in general, we count self-justification, dissonance resolution, confirmation bias, optimism and escapism among the possible goals. The tendency for people to selectively weight positive information and disregard negative self-referential information (as discussed in the Section entitled “Systematic Self-deception: Our Rose-tinted Glasses”) can be attenuated by interrupting cortical function in the left inferior frontal gyrus (Sharot et al., 2012). Belief updating is believed to engage multiple regions in the prefrontal cortex, but the left ventral PFC in particular appears to support the integration of new evidence with prior beliefs by tracking prediction errors, especially favorable surprises; negative information is selectively represented by the right ventral PFC, with differences in the strength of activation between people who tend to be optimistic or pessimistic, as discussed in the Section entitled “Systematic Self-deception: Our Rose-tinted Glasses” (Sharot et al., 2011). In addition to sensitivity for positive information, the left ventral PFC could serve an inhibitory function over the incorporation of negative information into the belief updating process. Such a positivity bias could plausibly serve as one mechanism supporting the ability to reason toward directionally motivated explanations, while also preserving the illusion of purely epistemic motivation due to the belief updating processes' still being implemented, albeit in an altered manner. This would support self-deception; it is plausible that cases of conscious, deliberate deception would involve either: (a) generation of at least two explanations, one of which we took to be epistemically the best, the other being the directionally-motivated one we actually give to others, or (b) simply giving an explanation we do not think is correct, but without thinking that we know the correct explanation.

### Motivated control of attention and memory

Neuroscience evidence suggests a role played by non-epistemic motivation states in controlling and supporting other cognitive functions, especially concerning memory retrieval, that may indirectly influence explanatory reasoning. Several experimental paradigms were developed specifically with the goal of demonstrating the differences in recall between items in memory that were intentionally remembered or willfully forgotten. Directed forgetting experiments attempt to disrupt memory encoding by instructing participants to remember or forget study stimuli immediately after their initial presentation (Fawcett and Taylor, 2008). Think/No-Think experiments instead allow participants to encode arbitrary relations between items by rehearsing them to a criterion of performance on tested recall, before instructing participants to block recall of the second item in a pair when presented with its paired “cue,” presumably to only disrupt the memory system at the retrieval phase before the testing phase (Anderson and Green, 2001). Each of the paradigms demonstrates the successful disruption of memory retrieval, while fMRI and electrophysiology evidence converge on the finding that right lateral PFC activation and left hippocampal deactivation are the neural markers of motivated forgetting (Anderson and Hanslmayr, 2014). The observed neural activations and behavioral effects are consistent with the thought suppression hypothesis of motivated forgetting, as shown in a memory-specific and hippocampus-targeted application of domain-general inhibitory control that is also supported by the right ventrolateral PFC (Anderson et al., 2004; Aron et al., 2004). It remains unclear whether thought suppression occurs by general inhibition of memory processing systems, or instead by the inhibition of specific memory traces; some have argued that current methods lack the behavioral specificity or spatial resolution in imaging to differentiate the two (Depue, 2012). However, it appears to us that motivated thinking in general, and motivated explanation in particular, do not involve a general inhibition of memory systems, but typically enhance memory for some information (that supporting a desired result) while inhibiting memory for other information. That is, in “trace” language, motivation effectively highlights some traces while leaving others at least temporarily in the dark.

Thought substitution is another mechanism of motivated forgetting, in which a memory targeted for forgetting is replaced by directing attention to another thought that can be paired with the original cue. Substitution, or competitive attention, is supported by different neural mechanisms than those supporting inhibition, due to its reliance on encoding and retrieving an alternative memory (Depue, 2012). Consistent with current views on directed retrieval, thought substitution engages both the left inferior frontal gyrus and the hippocampus (Anderson and Hanslmayr, 2014).

The findings on motivated forgetting are relevant to the discussion on motivated explanation in more than one way: retrieval of candidate explanations from storage in long-term memory is one step among many comprising inference to the best explanation under any motivation state; in addition, the process of confirming or disconfirming a candidate explanation that we may or may not want to accept involves, among other things, retrieval of confirming or disconfirming evidence. Processes that influence initial retrieval of explanations or of pertinent evidence would thus have downstream effects on any subsequent calculations in the brain, up to and including the final acceptance of an explanation as a confidently-held belief. Another possibility, however, is that thought substitution and inhibitory thought suppression are more extensive than can be inferred from the findings on memory alone. Thought substitution and suppression could thus enter the reasoning process twice: once at the level of memory retrieval, and again at the level of manipulating newly-encountered information, or information already being held in attention among competing explanations while reasoning to the best one. This would be an important factor, for example, in interpreting or reinterpreting propositions potentially relevant to the plausibility of explanations we are directionally motivated to accept or avoid. This possibility remains speculative until it can be tested directly—ideally, for situations in which motivation is more clearly defined and affective factors can be manipulated.

### Integrating the neuroscience evidence on motivated cognition

Together, the findings on the neural correlates of motivation and mechanisms of motivation-cognition interaction provide evidence for a tight coupling between non-epistemic motives and reasoning that includes both memory retrieval and the subsequent processing of current evidence in light of prior beliefs. This much is consistent with early views on the purely cognitive approach to studying motivated reasoning, which suggested a truncated or modified memory search for explanations as the driving mechanism (Kunda, 1990). However, directionally motivated reasoning may involve further processing systems as well. We have highlighted several cases of emotion-driven explanation, and also proposed a general motivation toward “emotional confirmation” parallel to the familiar cognitive confirmation bias. At present, however, it remains to be discovered exactly how motivation and reasoning systems in the brain interact with one another.

The flexibility of explanatory inference can be demonstrated at the behavioral level. Specifically, the plausibility of individual explanations is discounted when rival explanations with different mechanisms are simply considered at the same time, regardless of the respective explanations' individual plausibility (Sloman, 1994). This suggests that the plausibility of individual explanations is not “fixed,” then held constant during comparative evaluation; rather, credibility ratings of individual explanations can change, even without addition of any directly disconfirming evidence (a further question would be whether elimination of a competing explanation thereby raises the credibility of the remaining competitors). An additional implication is that even if the observed adjustments do not change comparative credibility ratings, they do constitute in effect a revision of our confidence in the “best” explanation. Perhaps further, if one considers an increasing number of at least moderately credible alternative explanations, one's credibility rating of the “best” explanation might sink to the point that one no longer regards it as the correct explanation, but only as a possibility that is somewhat more plausible than the rest. The neural mechanisms that would underlie either the actual behavioral results cited above, not to mention these further behavioral speculations, are far from clear. One can see nonetheless a plausible explanation of a familiar argumentative strategy: if we want to undercut someone's confidence in a particular explanation (e.g., when they postulate an unsavory intention behind someone's unwelcome behavior, or willful negligence on someone's part leading to a serious accident), we sometimes generate and propose reasonable alternative explanations. If we can produce multiple alternatives we may rightly say, “There are lots of other ways that could well have happened; you don't need to think anyone was negligent.” This would be an additional way in which directional motivation influences the generation of explanations and thereby influences judgments of credibility on candidate explanations. But again, this is simply a suggestion that calls for further investigation.

The neural mechanisms of motivated forgetting also warrant further exploration. Settling the distinction between general inhibition of memory systems or direct suppression of individual suppositions, as appears to occur routinely in directionally motivated explanation, appears to be as elusive as early attempts to search for the memory “engram” in the brain. And as with the search for an engram, even if we should discover the inhibition of a single memory trace, parallel to the discovery of visual processing neurons specific to a single face, we may be asking the wrong questions if the answers do not map onto human cognition in natural settings. Inhibition of memory traces or entire information processing systems in the bore of an MR machine may not have anything to do with forgetting, or selectively remembering, events that carry intense emotional value. Paradoxically, having reason to want to forget something, or to believe it, could even be revealed to preclude the ability to do so by means of “make-believe” or self-deception. To draw meaningful conclusions about how function in the brain supports mental function in daily life, it will be necessary to study memory for complicated personal events, and reasoning about explanations that are of consequence in several different sorts of ways to the person doing the reasoning.

We must remind ourselves that the current evidence concerning the neural correlates of motivated abductive reasoning is indirect, and will remain incomplete until direct evidence is available to evaluate the predictions suggested here. In addition, most other processes (besides motivated forgetting) involved in the generation and evaluation of explanations—along with the influence of motivation on those processes—remain even more in the dark.

### Future directions for studying motivated explanation in cognitive neuroscience

Much in the study of motivated explanation, and motivated thinking more generally, remains virtual *terra incognita*—starting with the processes involved in the generation of explanations. Most work in the philosophy of science and empirical psychology has focused either on normative criteria of explanation (what's epistemically “best”) or on sources of error due to a variety of widespread cognitive heuristics, biases, and other natural pitfalls. Much less is known about how motivation influences explanatory thinking, although we have been here able to draw on several helpful studies of explanation on the one hand, and of motivated thinking in general on the other. Our approach has been to set forth the multiple processes involved in generating and evaluating explanations, then to highlight specific types of explanatory motivation, indicating how they typically interact with those explanatory processes at the behavioral level. We predict that further study on the neural correlates of motivated explanation and reasoning will shed light on which aspects of explanation are susceptible to influence by motivational states. Specifically, we anticipate that the thought substitution and thought inhibition mechanisms that influence memory retrieval can be willfully applied during the reasoning process to help a person reach a desired conclusion. Furthermore, we expect the neural correlates of emotion and value to be activated by reasoning under conditions of directional motivation, although they would not be sufficient to influence the reasoning process without the engagement of executive control and attentional brain networks.

One approach to testing our hypotheses linking motivational states and the reasoning process is to have participants in a functional neuroimaging study perform some task involving a conflict between beliefs and evidence, thereby creating a conflict between the motivation to confirm prior beliefs and the epistemic motivation to know the truth. Yet another approach is to divide participants into groups who can be plausibly expected to respond differently to some emotionally-arousing or motivation-inducing visual stimuli, again while imaging their regional neural activations. Both of these experimental designs have been reported before, as reviewed in the current article. What remains to be discovered is the mechanism for motivation-reasoning interaction, and not simply a series of brain regions or a network whose activity corresponds to an interaction that can be assumed to be taking place. A first step toward uncovering the mechanisms for motivation-reasoning interaction will be to use a block-related imaging study design, in which successive experimental blocks in an fMRI protocol will selectively involve manipulations of the generation or evaluation of possible explanations in a reasoning task. If the possible explanations are generated by the experimenters for a participant, then the other behavioral degrees of freedom would involve evaluative components of reasoning, and vice-versa.

Finally, we expect real-time functional neuroimaging to be a useful tool in exploring the relationship between motivation and explanation in the brain. At the proof-of-concept level, it has been shown that by using the features of a motivationally-arousing visual stimulus as a feedback signal communicating a change in brain activity, study participants can learn to successfully modulate or inhibit brain activity in the networks corresponding to motivational arousal and visual cue reactivity (Sokunbi et al., 2014). If this could be applied in the reasoning context as well as in passive stimulus-viewing, then it may not be necessary to separate participants into groups who “should” be motivated a certain way by study stimuli. It may be similarly unnecessary to covertly manipulate participants' motivation to reach a particular conclusion or to create conflicts between belief and evidence. Instead, a vignette-based probabilistic reasoning task could be constructed with emotionally-arousing case materials, imaging brain activity as participants naturally respond to the stimuli while being instructed to alternately inhibit their emotions or respond freely. The successful self-regulation of emotion and motivational circuitry in the brain could then be used as a regressor predicting particular patterns of responses to the underlying probabilistic reasoning task.

It is plausible that behavioral and neuroimaging studies of priming effects (e.g., Schacter et al., 2004) could shed light on the effects of motivation. Insofar as one can distinguish the neural systems involved in classical priming studies from those involved in thought substitution and thought suppression, one could consider explanatory motivation as a type of primer, and a distinctive source of bias in memory search and in current perception of factors potentially supporting or disconfirming a preferred explanation.

## Conclusion

Our aim has been to demonstrate both the very wide scope and influence of motivation on the multiple stages and processes involved in the construction, evaluation and giving of explanations on the one hand, and the fact that most of this influence still awaits exploration on the other. Regarding the latter, we have made a number of suggestions for future study, both behavioral and neuroscientific.

We endorse the basic distinction drawn by others between epistemic and non-epistemic (directional) motivation, and have illustrated through concrete cases the way(s) in which motivations such as shifting blame, resolving cognitive dissonance, maintaining self-esteem, securing social harmony, and several more interact with explanatory processes and with various sorts of motivation to meet epistemic norms. At several points we have cited relevant work that is usually thought of in other terms, suggesting that it in fact can shed light on motivated explanation, and we have made specific suggestions along the way concerning future research.

We have emphasized throughout that finding and giving explanations is, like much else, a matter of meeting goals whose nature, timing, and urgency vary with circumstances, and that we very often have more than one motivation—some epistemic, some directional—for pursuing a particular explanation. These motivations can reinforce one another or they may conflict, since the explanation that best serves one purpose (e.g., securing social reconciliation) may or may not be the best explanation for other purposes (e.g., finding the most accurate explanation of someone's behavior). Where the two diverge we may succeed in finding a compromise solution, but we are sometimes motivated to find an explanation that serves one purpose at the expense of another. If so, our motivation may yet call for creating the appearance of meeting the sacrificed goal as part of a strategy for meeting our primary goal.

Finally, we emphasize once more the importance of emotion, and affect in general: it can powerfully drive any of the motivations, both epistemic and directional, considered here, and in fact is probably an element in all the directional motivations surveyed above (avoiding blame, bringing about reconciliation, etc.) Beyond that we have suggested that emotion generates its own source of explanatory bias, in that it motivates us to explain our emotion to ourselves, to understand our emotion, in a way that confirms it (shows it appropriate, rationalizes it). This “emotional confirmation bias” we take to be distinct from and parallel to the more familiar and better-studied cognitive confirmation bias concerning our beliefs. Moreover, affect probably is present to some extent even in the most “purely cognitive” effort to find an explanation, in the form of an evolved inclination to understand our environment in causal/explanatory terms and to receive at least a small reward when we think we achieve such understanding. In short, explanatory thinking cannot be understood without taking affect into account along with all the other processes that give rise to and constrain our explanatory thinking.

### Conflict of interest statement

The authors declare that the research was conducted in the absence of any commercial or financial relationships that could be construed as a potential conflict of interest.
